# The contribution of water contact behavior to the high *Schistosoma mansoni *Infection rates observed in the Senegal River Basin

**DOI:** 10.1186/1471-2334-11-198

**Published:** 2011-07-18

**Authors:** Seydou Sow, Sake J de Vlas, Foekje Stelma, Kim Vereecken, Bruno Gryseels, Katja Polman

**Affiliations:** 1 Prince Leopold Institute of Tropical Medicine, Antwerp, Belgium; 2 Department of Public Health, Erasmus MC, University Medical Center Rotterdam, The Netherlands; 3 Région Médicale de St Louis, St Louis, Senegal; 4 Department of Medical Microbiology, Virology Section, Radboud University Nijmegen Medical Centre, Nijmegen, The Netherlands

## Abstract

**Background:**

Schistosomiasis is one of the major parasitic diseases in the world in terms of people infected and those at risk. Infection occurs through contact with water contaminated with larval forms of the parasite, which are released by freshwater snails and then penetrate the skin of people. Schistosomiasis infection and human water contact are thus essentially linked, and more knowledge about their relationship will help us to develop appropriate control measures. So far, only few studies have related water contact patterns to infection levels.

**Methods:**

We have conducted detailed direct water contact observations in a village in Northern Senegal during the first years of a massive *Schistosoma mansoni *outbreak to determine the role of human water contact in the extent of the epidemic.

We quantified water contact activities in terms of frequency and duration, and described how these vary with age and sex. Moreover, we assessed the relationship between water contact- and infection intensity patterns to further elucidate the contribution of exposure to the transmission of schistosomiasis.

**Results:**

This resulted in over 120,000 recorded water contacts for 1651 subjects over 175 observation days. Bathing was the main activity, followed by household activities. Frequency and duration of water contact depended on age and sex rather than season. Water contacts peaked in adolescents, women spent almost twice as much time in the water as men, and water contacts were more intense in the afternoon than in the morning, with sex-specific intensity peaks. The average number of water contacts per person per day in this population was 0.42; the average time spent in the water per person per day was 4.3 minutes.

**Conclusions:**

The observed patterns of water contact behavior are not unusual and have been described before in various other settings in sub-Saharan Africa. Moreover, water contact levels were not exceptionally high and thus cannot explain the extremely high *S. mansoni *infection intensities as observed in Northern Senegal. Comparison with fecal egg counts in the respective age and sex groups further revealed that water contact levels did not unambiguously correspond with infection levels, indicating that factors other than exposure also play a role in determining intensity of infection.

## Background

In the late 1980s, Northern Senegal was confronted with a severe outbreak of *Schistosoma mansoni *infection after the construction of a dam on the Senegal River and subsequent water resource development. In a few years, the prevalence in Ndombo, the epicenter of the epidemic, rose from 0% (non existing) before 1988 to 75-100% in 1992, with the highest intensities of infection ever described world-wide [1,2]. These were attributed to intense transmission and the supposed lack of acquired immunity in this recently exposed community. Epidemiological studies in four successive cohorts, however, showed that infection intensities had similar age-related patterns as in conventional endemic situations in sub-Saharan Africa, i.e. with egg counts and antigen levels increasing to a peak in adolescents and strongly declining in adults [1,3]. This would leave age-related exposure differences as the most obvious explanation for the observed patterns. As the available water contact data at that time did not support this possibility, alternative explanations of age-specific mechanisms other than acquired immunity or exposure were put forward, such as skin permeability or hormonal factors [2,4,5]. Nevertheless, important questions on the role of exposure remain to be answered. It is still unclear if and to what extent the observed extremely high infection levels in Northern Senegal were due to intense exposure, and in how far the endemic-like age-related infection patterns were due to differences in exposure levels, measured by human water contact patterns as a proxy for true exposure.

Many studies have attempted to measure individuals' exposure, either by directly observing behavior at water sources [6-18], or indirectly by interview/questionnaire [19-27]. Only few studies attempted to quantify variables that are important in determining age and sex patterns of exposure from the corresponding patterns of water contact [12,16,28-31] and even less tried to relate these to infection levels and patterns [5,11,32,33].

We have maintained detailed direct water contact observations in Ndombo during the first years of the *S. mansoni *epidemic in Northern Senegal. The analysis of this unique dataset, amounting to over 120,000 recorded contacts, is presented here. We quantified water contact activities in terms of frequency and duration, and described how these vary with age and sex. In this way, we aimed to determine whether water contact patterns in Ndombo were exceptional or comparable to those in traditional schistosomiasis endemic communities in sub-Saharan Africa. Moreover, we assessed the relationship between water contact- and infection intensity patterns to further elucidate the contribution of exposure to the transmission of schistosomiasis.

## Methods

### Study site and population

**Figure 1:**
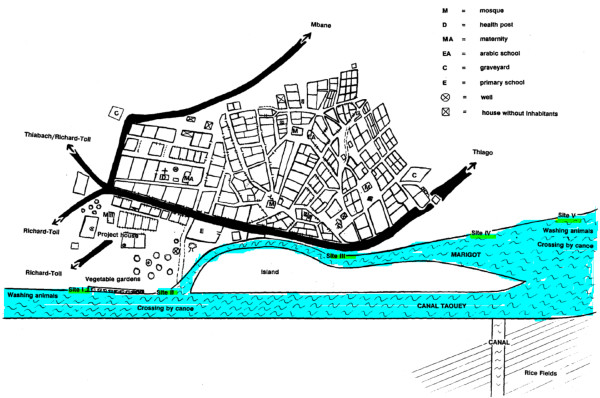
**Main water contact sites in the village of Ndombo, Northern Senegal**.

The study took place in Ndombo, a village situated 3 km south of the city of Richard Toll in the Delta of the Senegal River Basin (Northern Senegal). The village counts about 3000 inhabitants, mostly Wolof ethnic group of Muslim faith. The majority of the population works in rice farming, small-scale market gardening, fishing, or is employed in the nearby sugar cane estate. The climate in the area is arid and characterized by a hot dry season with temperatures up to 45°C (April to June), a hot and humid period (July to October), and a relatively cold dry season between November and April in which temperatures can drop to 10-15°C. The village is situated along a man-made canal and marshland (Taouey) that connects the sugar cane plantations, the Senegal River, and an inland lake. The population depends largely on this water source for domestic, recreational as well as occupational purposes, and most water contact activities take place at five well-defined sites on the banks of the canal and the marshland (Figure 1).

This study is part of a larger investigation on schistosomiasis epidemiology, transmission and control in Senegal, for which approval was obtained from the ethical committees of the Leiden University Medical Centre, the Netherlands, the Institute of Tropical Medicine in Antwerp, Belgium, and the Ministry of Health in Dakar, Senegal, respectively.

### Data collection

Direct observations of individual water contacts were carried out from September 1991 to September 1993 (25 months). The sites selected for the study were the main water contact sites in the village where most activities leading to schistosomiasis infection occurred (see Figure 1). Twelve observers with formal education were chosen among the villagers and submitted to one week of training before the start of the study. The water contact behavior of the whole population of Ndombo was observed from 6 a.m. till 7 p.m. (13 hours) seven days each month. For each site, the observers were divided into pairs. The first shift with the first observer was from 6 to 12.30 a.m., the second from 12.30 to 7 p.m. For the most crowded site (site II in Figure 1), two teams of observers were selected, whereby one team observed the males and the other team observed the females. A local supervisor randomly visited the observers several times per observation day to make sure that the observations were accurate and standardized.

Each individual entering the water was identified by the observer and recorded in a notebook by name, age, sex, type of water contact activity, time of entrance into, and exit out of the water. Nine different types of activities were recorded: a] *(Dis)embarking*: embarking or disembarking a boat to cross the stream; b] *Small bath*: small bath, ablution and/or drinking; c] *Bathing*: bathing with or without soap, swimming and/or playing; d] *Collecting water*: fetching water for domestic purposes; e] *Household*: doing laundry and/or dishes; f] *Animals*: watering and/or washing animals in the water; g] *Private toilet*: washing of genitals, bottom; h] *Fishing*: fishing related activities in or near the water; i] *Irrigation*: irrigation and/or removing vegetation from the water. If a range of activities, i.e. more than one type of activity, took place between time of entrance into the water and exit out of the water, these were noted down consecutively, and marked as combined activity. Among the water contact recordings of the total population, only those individuals belonging to the four studied cohorts were considered in our study. These cohorts consisted of random population samples of approximately 400 subjects each, selected at 8-month intervals between 1991 and 1994, and examined for *S. mansoni *infection by egg counts in stools, using the Kato-Katz method [1,2]. Their corresponding individual codes were recorded in the notebooks retrospectively. The data were entered daily into a Microsoft Excel database, and extensively checked for errors, suspicious values and outliers.

### Data analysis

**Figure 2:**
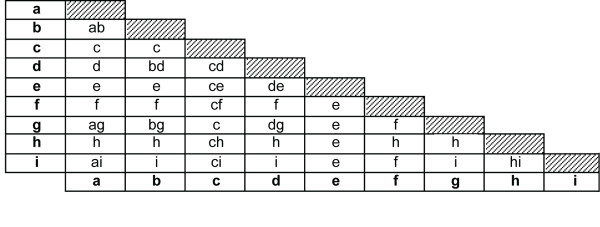
**Decision triangle on combined water contact activities**. Decision triangle illustrating how combined water contact activities were dealt with. The left column and lowest line indicate single activities (bold). The corresponding cell represents the decision made: (1) one of both activities was considered dominant (only the dominant activity is given in the cell); or (2) both activities were considered relevant (both are given). In the latter situation, for calculations of durations per activity (Table 2 and Figure 3), the combined activity was split proportionally to the average durations of the single activities (upper half of Table 1). All combinations were treated symmetrically, i.e. the decision about 'a + b' and 'b + a' was the same. Combinations of three activities followed the same procedure. For example, 'a + b + c' results in 'c', and 'g + h + i' results in 'hi'. The meaning of the codes for the nine single activities is given in Table 1.

Two exposure indices were used, frequency and duration of water contact. Frequency was defined by the number of water contacts, irrespective of the (type of) activity. A range of two or more observed activities was considered as one water contact. Table 1 lists the single and combined activities which were used to determine frequencies. Duration was defined by the time spent in the water during a water contact activity. In case of a combination of two or more observed activities the most dominant was chosen, based on degree of exposure and/or duration of contact, or they were split up when one was not clearly dominant over the other (Figure 2). Table 2 shows the resulting total and average durations of activities.

**Table 1:** Overview of all 121,771 observed water contacts of the 1,651 members of four epidemiological cohorts in Ndombo, Northern Senegal, during 175 days (7 days in each of 25 successive months) of observations; first the frequency and duration of all nine single water contact activities are given, followed by the most frequently observed combined activities

Activity	Description	Total duration (min)	Total count	Average duration (min)
a	(Dis)embarking	8858	4407	2.0
b	Small bath	11781	3670	3.2
c	Bathing	617543	54844	11.3
d	Collecting water	55659	29141	1.9
e	Household	91404	3282	27.9
f	Animals	13353	1215	11.0
g	Private toilet	510	216	2.4
h	Fishing	9353	383	24.4
i	Irrigation	11979	1572	7.6
*Subtotal*		*820439*	*98730*	*8.3*
a + b	(Dis)embarking + small bath	957	368	2.6
b + d	Small bath + collecting water	5018	945	5.3
b + g	Small bath + private toilet	368	102	3.6
c + d	Bathing + collecting water	213561	14285	15.0
c + d + e	Bathing + collecting water + household	6711	203	33.1
c + e	Bathing + household	144252	3740	38.6
c + f	Bathing + animals	18889	1221	15.5
c + h	Bathing + fishing	2440	81	30.1
c + i	Bathing + irrigation	10331	483	21.4
d + e	Collecting water + household	34494	1453	23.7
d + g	Collecting water + private toilet	317	120	2.6
Other	Combined activities with < 50 observations	208	40	5.2
*Subtotal*		*437546*	*23041*	*19.0*
Total	All activities	1257985	121771	10.3
	Per individual	762	74	
	Per individual per day	4.4	0.42	

**Table 2:** Overview of the total and average durations of the nine types of water contact, after processing of the combined activities

Activity	Description	Total duration (min)	Total count	Average duration (min)
a	(Dis)embarking	9255	4802	1.9
b	Small bath	15755	5100	3.1
c	Bathing	859996	74857	11.5
d	Collecting water	91184	46158	2.0
e	Household	230961	8678	26.6
f	Animals	22683	2436	9.3
g	Private toilet	882	473	1.9
h	Fishing	11076	466	23.8
i	Irrigation	16194	2060	7.9
Total	All activities	1257985	145015	8.7
	Per individual	762	88	
	Per individual per day	4.4	0.50	

See Table 1 for the number of subjects and observation time.

Frequencies and durations of water contact data were further categorized per age group (0-9 years, 10-19 years, more than 20 years old) and sex. They were divided according to season (hot wet, hot dry, and cold dry), time of the day (divided into hourly intervals) and type of activity (see above). These exposure indices were averaged and related to intensity of infection, as expressed by the arithmetic mean eggs per gram feces (EPG), both pre-control and measured 1 year after treatment. Infection intensities (EPG pre-control and one year after treatment) for the six age/sex-categories were plotted against frequency and duration of water contact, and values of R-squared were calculated, assuming a proportional association. Also, water contact values were adjusted for body surface (percentage of the body exposed related to a certain water contact activity, with a maximum of 100% for 'bathing') and time of day (reflecting the mean relative number of cercariae assumed to be in the water during that time, with a peak between 1 p.m. and 2 p.m. - assuming that 0.1% of cercariae were viable after 24 h). These adjusting factors have previously been applied by Scott *et al*., who tested various assumptions regarding the relationship between water contact and exposure in a questionnaire-based study in Northern Senegal [5]. An improvement of the fit was determined by the percentage reduction of the residual variance (unexplained variation) for the model with adjustment as compared to the one without.

## Results

The number of recorded contacts was 121,771 (Table 1) with 1,257,985 minutes of observed water contact spread over 2 years (September 1991 - September 1993). Bathing/swimming appeared to be the main activity, both in terms of duration and frequency, followed by household activities. Some categories took relatively long, but occurred rarely, such as fishing; for other activities, such as collecting water, it was the other way around (Table 2). Fishing, (dis)embarking, small bath, animals, private toilet and irrigation played a negligible role, both in terms of frequency and duration.

**Figure 3:**
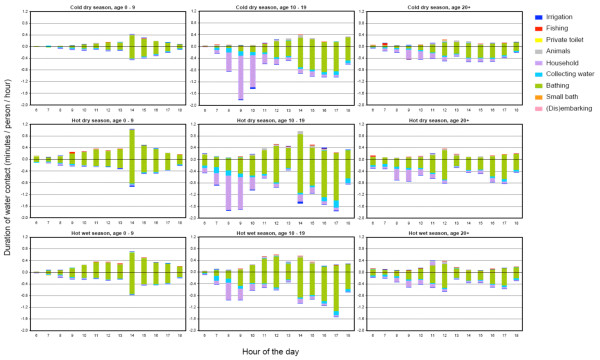
**Duration of water contact**. Duration of water contact by season, hour of the day, age, sex (boys up = positive values; girls down = negative values), and type of activity (see legend) for 1,651 inhabitants of the rural village of Ndombo in Northern Senegal as recorded during 175 days of observational studies. This graph reflects the division of the total durations (i.e. 1,257,985 min) given in Table 2. Hour of the day is the first moment of each one-hour interval.

**Figure 4:**
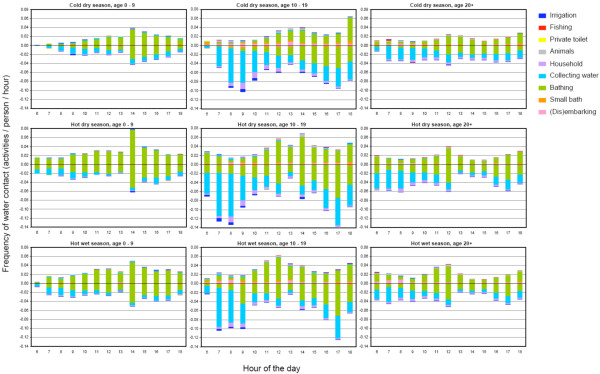
**Frequency of water contact**. Frequency of water contact by season, hour of the day, age, sex (boys up = positive values; girls down = negative values), and type of activity (see legend) for 1,651 inhabitants of the rural village of Ndombo in Northern Senegal as recorded during 175 days of observational studies. This graph reflects the division of the 121,771 total frequencies given in Table 1, where combinations of two or three activities were attributed to the corresponding single activities with weight of 0.50 and 0.33, respectively. Hour of the day is the first moment of each one-hour interval.

Figure 3 and 4 show the duration and frequency of water contact per season, age and sex. Although in the hot dry season water contacts were slightly more intense than for the other seasons, patterns of both duration and frequency depend much more on age than on the season. In the age group of 10-19 years, longer and more frequent water contact was observed than in the other age groups. Women showed substantially more and longer water contact than men; for the older age groups (10-19 and more than 20 years), duration and frequency of water contact in women were even twice that of men. Female adolescents (10-19 years) showed the most intense water contact, which is mainly due to collecting water more often and a longer time spent on household activities. Most intense water contact occurred between noon and 2 p.m. for men; for women two distinct peaks were observed, one between 8 and 10 a.m., and one between 2 and 5 p.m.

**Figure 5:**
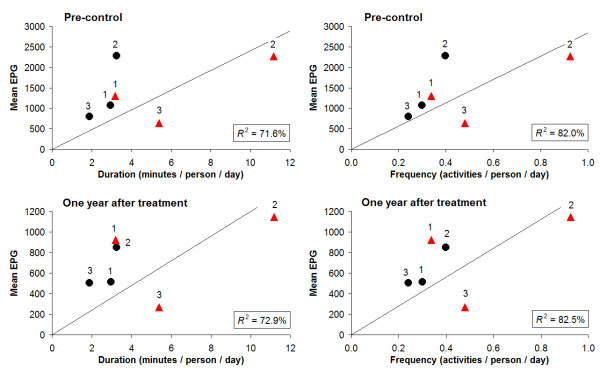
**Infection intensity in relation to duration and frequency of water contact**. Infection intensity (arithmetic mean EPG, both pre-control and one year after treatment) in relation to duration and frequency of water contact, for six demographic groups based on three age categories and both sexes. The durations are the accumulation over season, hour of the day, and type of activity of the values for each demographic group in Figure 3. Similarly, the frequencies are the accumulation of values in Figure 4. Black dots = males, red triangles = females; 1 = 0-9 years, 2 = 10-19 years, 3 = 20 years and above.

Figure 5 shows for each age- and sex group the infection intensity in relation to duration and frequency of water contact. It is clear that EPG-values (both pre-control and 1 year after treatment) are not linearly related with water contact. Especially adult females had lower egg counts than would be expected from the duration and frequency of their water contacts, while male adolescents appeared to have the highest EPG/water contact ratio. The average number of water contacts per person per day in this population was 0.42; the average time spent in the water per person per day was 4.3 minutes.

**Figure 6:**
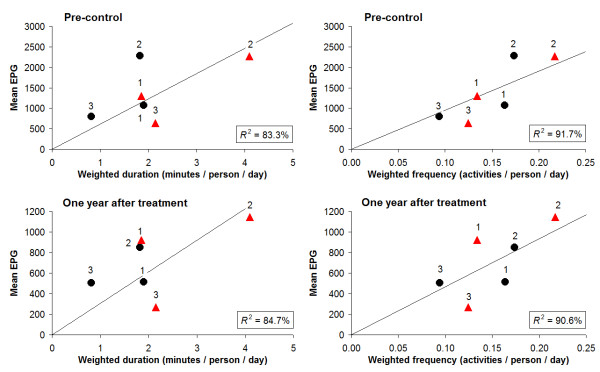
**Infection intensity in relation to 'weighed' duration and frequency of water contact**. Similar as Figure 5, but then adjusted for body surface and time of day (reflecting cercarial density), as previously described by Scott *et al*. [5]. As compared to the unadjusted model (Figure 5), the residual variance (unexplained variation) of the adjusted model reduced by 41% (duration) and 54% (frequency) pre-control, and 44% (duration) and 46% (frequency) one year after treatment, respectively.

Figure 6 shows for each age- and sex group the infection intensity in relation to duration and frequency of water contact, after adjustment for body surface and time of day of contact. Both proxies for exposure, especially frequency, showed a better fit with intensities of infection when adjusting for these two factors (Figure 6 vs Figure 5). Nevertheless, the relationship between infection and water contact patterns still deviated from a clear proportional one, especially in women.

## Discussion

After the outbreak of a *S. mansoni *epidemic, extremely high infection rates and typical endemic-like age-related infection patterns were observed in Ndombo, Senegal. The main justification of the current study was to determine the contribution of water contact behavior to these exceptional observations. Descriptive analysis of the water contact activities showed that bathing was the main activity, followed by household activities; water contacts peaked in adolescents; women spent almost twice as much time at the water than men; water contacts were more intense in the afternoon than in the morning, with sex-specific intensity peaks; and frequency and duration of water contact depended on age and sex rather than season. These findings are not unusual and have been described before in various other settings [7,10,16,30,33-36]. Since infected snails were attested to be present throughout the year [37] and marked seasonal variations of water contact behavior appeared to be absent, transmission in this area is likely to be perennial.

Also in terms of the number of water contacts per person per day, our results in Northern Senegal did not show marked differences with other schistosomiasis endemic countries where intensive observational water contact studies have been performed. Indeed, our finding of an average of 0.42 contacts per person per day is within the range of Fulford *et al*. [10], who reported a mean annual frequency of 12.8 to 162 water contacts/person among seven *S. mansoni *endemic communities in Kenya, which is 0.04 to 0.44 contacts/person/day (median 0.17). Exact average water contact durations for these communities were not reported, but from the graphs it can be deduced that this was about 880 minutes/person annually, and thus 2.4 minutes per day. Again, this is of the same order of magnitude as the average duration of 4.3 minutes spent in the water per day as presented here for Northern Senegal. Chandiwana & Woolhouse [38] reported a mean rate of water contact of 0.43 contacts/person/day (ranging from zero to 3.3 contacts/person/day), in a *S. haematobium *endemic area in Zimbabwe, which is remarkably similar to our values for Senegal. Other water contact studies based on direct observations used different exposure indices, which precludes straightforward comparison [32,39,40]. Nevertheless, from the available observational water contact studies we can conclude that the water contact levels in Ndombo are not exceptionally high and thus cannot explain the extremely high infection intensities in this area as compared to the other studies.

Both infection- and water contact patterns in this community were found to be clearly age-and sex-related. Looking at these patterns more closely however, we found that in the respective age- and sex groups more or longer water contact did not unequivocally lead to high infection intensities (Figure 5 +6). In the oldest two age groups, women showed substantially more and longer water contact than men, while infection levels were comparable. An explanation for this finding could be that male water-related activities entail relatively intense water contact. Indeed, the main male activity was bathing, which can be considered a more risky behaviour in terms of body exposure as compared to typical female activities such as collecting water or doing laundry/dishes. Also the degree of using soap, which may have a 'cercaricidal' effect [10], for washing clothes and/or bathing may have influenced infection intensities in women and men in different ways. Moreover, in men most intense water contact occurred between noon and 2 p.m., the part of the day when cercarial emission has been reported to be highest in the Senegal River Basin [41], while women's water contact peaked in the morning and afternoon. Indeed, after adjusting for body surface and time of day, the association between water contact and intensities of infection appeared to be stronger than without including these factors, but differences in this relationship across the demographic groups remained (Figure 6). In addition, male adolescents showed much higher infection levels than the other male age groups, while they did not bath significantly more or longer than young or adult men, nor at different times of the day. Another hypothetical explanation may be that certain typical male activities have not been captured, as these may have taken place outside the designated observation time or sites (e.g. fishing). However, we have no indications for such an effect in males only.

Despite observed differences in infection- and water contact patterns depending on age and sex, exposure appears not to be the only factor of importance in determining intensity of infection before or one year after treatment in this *Schistosoma *epidemic focus. Similar conclusions resulted from studies in stable endemic situations [11,39,42,43]. A few have found some relationship between water contact and infection intensity, although not very strong/convincing [12,38,44]. It should be noted that for any of these studies, including ours, it cannot be excluded that exposure factors other than those taken into account, may have somehow contributed to the observed age- and sex-related differences in infection levels [5,39]. In the specific case of Ndombo, very high numbers and infection rates of *Biomphalaria pfeifferi*, the snail intermediate host of *S. mansoni*, were found at the start of the epidemic [2], which could also have (partly) accounted for the extremely high *S. mansoni *infection intensities of the community. An obvious alternative explanation other than exposure would be the absence of acquired immunity in this recently exposed, supposedly non-immune community, but this does not correspond with the observed endemic-like age-related infection patterns in this community [1,3].

For women, the ratio of infection intensity to water contact (i.e. the slope of a hypothetical line from the origin to a point) in Figure 5 and - to a lesser extent - Figure 6 decreased with age, suggesting an increasing degree of resistance with age. In an epidemic focus like Ndombo, immunity should not, or at most partially, have developed at the time of the data collection. Thus, this resistance is more likely to be due to some other age-related, innate factor [2,4,5]. In men, however, this pattern seemed to be absent, with the youngest age group showing a slightly lower ratio than the older groups, particularly after adjusting for body surface and time of day (Figure 6). As yet, there is no biological evidence that could explain why such resistance would only occur in adult women and not in adult men. Post-pubertal hormonal or other (both age- and sex-related) factors may play a role. For example, Fulford *et al*. [45] suggested that gonadal steroids affecting the immune system may lie behind the common observation, originally made by Butterworth *et al*. [46], that women are usually infected less heavily than men yet generally have more water contact.

A non-proportional relationship between egg counts and water contact may also indicate that egg production is suppressed in individuals with higher water contact, and thus suggesting some form of density-dependence. For example, density-dependent fecundity has been widely implicated as a major regulatory force in maintaining helminth population stability, and is generally considered to result either from competition between parasites for host resources or from immunological control [47]. The phenomenon is still controversial for schistosomiasis [48-54]. And yet again, we cannot explain why such density-dependence would only occur in women and not in men.

To our knowledge, this is the largest dataset published so far of directly observed recorded water contacts, spanning a two year time period in the early years of an *S. mansoni *epidemic. Direct water contact observations have more quantitative and qualitative value than water contact information based on questionnaires, which are easy to perform but have inherent well-known limitations such as overreporting, recall bias and information bias [55,56]. This is illustrated by a questionnaire-based study in four villages in the same *S. mansoni *affected area as Ndombo [5]. This study reported a mean of 4.4 water contacts per day with a median duration of 57 min per day, which is in sharp contrast with the relatively low numbers found in the present study. It has been noted before that levels and patterns of contact can vary dramatically between culturally similar communities, and even within a single village [10,36], but these extreme numbers are more likely to be due to the way the water contact information was collected [56].

A few limitations are present in this study. All water contact measurement tools, including direct water contact observations are only an indirect measure of true exposure, i.e. exposure to infectious cercariae. It is impossible to determine true exposure [10]. Different authors have approximated and compared exposure from water contact behavior in various ways [5,10,32,34,39,57-59], but the possibility will always remain that their and our conclusions are based on an inadequate understanding of how water contact translates into exposure. Moreover, direct water contact observations may be subject to 'observer effect', occurring when subjects alter their behaviour because an observer is present. For example, we did not observe a single act of direct defecation into the stream, even though such an activity would occasionally be expected, at least for children, to explain the intense transmission of *S. mansoni *in this area [60]. Also, direct observation tends to underreport water contacts taking place outside the limits of observation time or of observation sites [30]. For that reason, some fishing-related contacts may have gone unnoticed, as fishing by its nature is much dispersed [36]. The same holds for occupational activities in the adjacent rice and sugar cane fields. Last but not least, exposure does not depend on human behaviour alone, and should ideally be analysed in relation to snail studies or cercariometry.

## Conclusion

The water contact levels in Ndombo are not exceptionally high and thus cannot explain the extremely high infection intensities as observed in this area during the *S. mansoni *epidemic. Absence of an effective acquired immunity could play a role, but this is in contrast with the strongly decreasing ratio of infection intensity/water contact with age that we found for women, as well as with previous immuno-epidemiological studies in the same area [1,3]. Although both infection- and water contact patterns in this community were found to be age- and sex-related, there is no clear proportional relationship between exposure and infection intensity, neither before nor one year after treatment in this *Schistosoma *epidemic focus. The finding that water contact and infection levels do not unambiguously correspond with each other, irrespective of the assumptions made regarding the relationship between water contact behaviour and exposure, indicates that in this population other factors than only exposure play an important role in determining intensity of infection. Further research is needed, with respect to the translation of water contact data into actual exposure, as well as the relation between exposure and actual *Schistosoma *infection at the individual level.

## Competing interests

The authors declare that they have no competing interests.

## Authors' contributions

SS contributed to the design of the study, participated in the data collection and data analysis, and helped to draft the manuscript. SJDV designed and coordinated the data analysis, and drafted the manuscript. FS participated in the design and coordination of the study, and revised the manuscript. KV participated in the data analysis and the revision of the manuscript. BG conceived of the study, participated in its design and coordination, and revised the manuscript. KP contributed to the design and analysis of the data, and drafted the manuscript. All authors read and approved the final manuscript.

## Pre-publication history

The pre-publication history for this paper can be accessed here:

http://www.biomedcentral.com/1471-2334/11/198/prepub

## References

[B1] Stelma FF Talla I Polman K Niang M Sturrock RF Deelder AM Gryseels B Epidemiology of *Schistosoma mansoni *infection in recently exposed community in Northern Senegal Am J Trop Med Hyg 1993 49 701 706 8279638 10.4269/ajtmh.1993.49.701

[B2] Gryseels B Stelma FF Talla I van Dam GJ Polman K Sow S Diaw M Sturrock RF Doehring-Schwerdtfeger E Kardorff R Decam C Niang M Deelder AM Epidemiology, immunology and chemotherapy of *Schistosoma mansoni *infections in a recently exposed community in Senegal Trop Geogr Med 1994 46 209 219 7825223

[B3] Polman K Stelma FF Gryseels B Van Dam GJ Talla I Niang M Van Lieshout L Deelder AM Epidemiological application of circulating antigen detection in a recent *Schistosoma mansoni *focus in northern Senegal Am J Trop Med Hyg 1995 53 152 157 7677216 10.4269/ajtmh.1995.53.152

[B4] Polman K Stelma FF Le Cessie S De Vlas SJ Falcão Ferreira ST Talla I Deelder AM Gryseels B Evaluation of the patterns of *Schistosoma mansoni *infection and re-infection in Senegal, from faecal egg counts and serum concentrations of circulating anodic antigen Ann Trop Med Parasitol 2002 7 679 689 10.1179/000349802125001708 12537629

[B5] Scott JT Diakhaté M Vereecken K Fall A Diop M Ly A De Clercq D De Vlas SJ Berkvens D Kestens L Gryseels B Human water contacts patterns in *Schistosoma mansoni *epidemic foci in northern Senegal change according to age, sex and place of residence, but are not related to intensity of infection Trop Med Int Health 2003 8 100 108 10.1046/j.1365-3156.2003.00993.x 12581433

[B6] Bundy DAP Blumenthal UJ Barnard CJ, Behnke JM Human behavior and the epidemiology of helminth infections: the role of behavior in exposure to infection Parasitism and Host Behavior 1990 Taylor & Francis: London-New York-Philadelphia 364 389

[B7] Coulibaly G Diallo M Madsen H Dabo A Traoré M Keita S Comparison of schistosome transmission in a single - and a double - cropped area in the rice irrigation scheme, 'Office du Niger', Mali Acta Trop 2004 91 15 25 10.1016/j.actatropica.2004.02.008 15158685

[B8] Dalton PR Pole D Water-contact patterns in relation to *Schistosoma haematobium *infection Bull World Health Organ 1978 56 417 426 308406 PMC2395583

[B9] El Katsha S Watts S Schistosomiasis in two Nile delta villages: an anthropological perspective Trop Med Int Health 1997 2 846 854 10.1046/j.1365-3156.1997.d01-409.x 9315043

[B10] Fulford AJ Ouma JH Kariuki HC Thiongo FW Klumpp R Kloos H Sturrock RF Butterworth AE Water contact observations in Kenyan communities endemic for schistosomiasis: methodology and patterns of behaviour Parasitology 1996 113 223 241 10.1017/S0031182000082007 8811848

[B11] Kabatereine NB Vennervald BJ Ouma JH Kemijumbi J Butterworth AE Dunne DW Fulford AJ Adult resistance to schistosomiasis mansoni: age-dependence of reinfection remains constant in communities with diverse exposure patterns Parasitology 1999 118 101 105 10.1017/S0031182098003576 10070667

[B12] Kloos H Higashi GI Cattani JA Schlinski VD Mansour NS Murrel KD Water contact behavior and schistosomiasis in an upper Egyptian village Soc Sci Med 1983 17 545 562 10.1016/0277-9536(83)90297-6 6879254

[B13] Kvalsvig JD Schutte CHJ The role of human water contact patterns in the transmission of schistosomiasis in an informal settlement near a major industrial area Ann Trop Med Parasitol 1986 80 13 26 3089185 10.1080/00034983.1986.11811980

[B14] Ofoezie JE Christensen NO Madsen H Water-contact patterns and behavioral knowledge of schistosomiasis in south-west Nigeria J Biosoc Sci 1998 30 245 259 10.1017/S0021932098002454 9746827

[B15] Tayo MA Pugh RNH Bradley AK Mulumfashi endemic diseases research project, XI. Water-contact activities in the schistosomiasis study area Ann Trop Med Parasitol 1980 74 347 354 7396567 10.1080/00034983.1980.11687351

[B16] Watts S Khallaayoune K Bensefia R Laamrani H Gryseels B The study of human behavior and schistosomiasis transmission in an irrigated area in Morocco Soc Sci Med 1998 46 755 765 10.1016/S0277-9536(97)00171-8 9522434

[B17] World Health Organization Workshop on the role of human-water contact in schistosomiasis transmission St Lucia 1979 TDR/SER-HWC/79.3

[B18] Woolhouse ME Etard JF Dietz K Chandiwana SK Hagan P Heterogeneities in schistosome transmission dynamics and control Parasitology 1998 117 475 482 10.1017/S003118209800331X 9836312

[B19] Barbosa CS Barbosa FS Schistosomiasis epidemiological patterns in a community of small farmers in Pernambuco State, Brazil Cad Saude Publica 1998 14 129 137 10.1590/S0102-311X1998000100020 9592218

[B20] Barreto ML Use of risk factors obtained by questionnaires in the screening for *Schistosoma mansoni *infection Am J Trop Med Hyg 1993 48 742 747 8333567 10.4269/ajtmh.1993.48.742

[B21] Coura-Filho P Rocha RS Farah MW da Silva GC Katz N Identification of factors and groups at risk of infection with *Schistosoma mansoni*: a strategy for the implementation of control measures? Rev Inst Med Trop Sao Paulo 1994 36 245 253 7855489 10.1590/s0036-46651994000300009

[B22] Da Silva AA Cutrim RN de Brittoe Alves MT Coimbra LC Tonia SR Borges DP Water contact patterns and risks factors for *Schistosoma mansoni *infection in a rural village of northern Brazil Rev Inst Med Trop Sao Paulo 1997 39 91 96 9394521 10.1590/s0036-46651997000200005

[B23] Lima e Costa MF Magalhães MH Rocha RS Antunes CM Katz N Water contact patterns and socio-economic variables in the epidemiology of schistosomiasis mansoni in an endemic area in Brazil Bull World Health Organ 1987 65 57 66 3107847 PMC2490852

[B24] Lima e Costa MF Rocha RS Firmo JO Guerra HL Passos VA Katz N Questionnaires in the screening for *Schistosoma mansoni *infection: a study of socio demographic and water contact variables in four communities in Brazil Rev Inst Med Trop Sao Paulo 1998 40 93 99 9755562 10.1590/s0036-46651998000200005

[B25] Moza PG Pieri OS Barbosa CS Rey L Socio-demographic and behavioral factors related to schistosomiasis in a rural village of the sugar cane belt in Pernambuco State, Brazil Cad Saude Publica 1998 14 107 115 9592216 10.1590/s0102-311x1998000100018

[B26] Firmo JO Lima Costa MF Guerra HL Rocha RS Urban schistosomiasis morbidity, socio-demographic characteristics and water contact patterns predictive of infection Int J Epidemiol 1996 25 1292 1300 10.1093/ije/25.6.1292 9027538

[B27] Ximenes RA Southgate B Smith PG Guimaraes Neto L Social environment, behavior and schistosomiasis in an urban population in northern Brazil Rev Panam Salud Publica 2001 9 13 22 11253273 10.1590/s1020-49892001000100005

[B28] Brinkmann UK Korte R Schmidt-Ehry B The distribution and spread of schistosomiasis in relation to water resources development in Mali Trop Med Parasitol 1988 39 182 185 3140361

[B29] Chandiwana SK Seasonal patterns in water contact and the influence of water availability on water contact activities in two schistosomiasis endemic areas in Zimbabwe Cent Afr J Med 1987 33 8 15 3690652

[B30] Gazzinelli A Bethony J Fraga LA LoVerde PT Corréa-Oliveira R Kloos H Exposure to *Schistosoma mansoni *infection in a rural area of Brazil. I: water contact Trop Med Int Health 2001 6 126 135 10.1046/j.1365-3156.2001.00684.x 11251909

[B31] Bethony J Williams JT Kloos H Blangero J Alves-Fraga L Buck G Michalek A Williams-Blangero S LoVerder PT Corréa-Oliveira R Gazzinelli A Exposure to *Schistosoma mansoni *infection in a rural area in Brazil. II. household risk factors Trop Med Int Health 2001 6 136 145 10.1046/j.1365-3156.2001.00685.x 11251910

[B32] Woolhouse ME Mutapi F Ndhlovu PD Chandiwana SK Hagan P Exposure, infection and immune responses to *Schistosoma haematobium *in young children Parasitology 2000 120 37 44 10.1017/S0031182099005156 10726264

[B33] Rudge JW Stothard JR Basáñez MG Mgeni AF Khamis IS Khamis AN Rollinson D Micro-epidemiology of urinary schistosomiasis in Zanzibar: Local risk factors associated with distribution of infections among schoolchildren and relevance for control Acta Trop 2008 105 45 54 10.1016/j.actatropica.2007.09.006 17996207

[B34] Kloos H Rodrigues JC Pereira WR Velásquez-Meléndez G Loverde P Corréa- Oliveira R Gazzinelli A Combined methods for the study of water contact behaviour in a rural schistosomiasis-endemic area in Brazil Acta Trop 2006 97 31 41 10.1016/j.actatropica.2005.08.006 16212926

[B35] Ndassa A Mimpfoundi R Gake B Paul Martin MV Poste B Risk factors for human schistosomiasis in the Upper Benue valley, in northern Cameroon Ann Trop Med Parasitol 2007 101 469 477 17716429 10.1179/136485907X193752

[B36] Pinot de Moira A Fulford AJ Kabatereine NB Kazibwe F Ouma JH Dunne DW Booth M Microgeographical and tribal variations in water contact and *Schistosoma mansoni *exposure within a Ugandan fishing community Trop Med Int Health 2007 12 724 735 10.1111/j.1365-3156.2007.01842.x 17550469

[B37] Sturrock RF Diaw OT Talla I Niang M Piau JP Capron A Seasonality in the transmission of schistosomiasis and in populations of its snail intermediate hosts in and around a sugar irrigation scheme at Richard Toll, Senegal Parasitology 2001 123 77 89 11769294 10.1017/s0031182001008125

[B38] Chandiwana SK Woolhouse ME Heterogeneities in water contact patterns and the epidemiology of *Schistosoma haematobium* Parasitology 1991 103 363 370 10.1017/S0031182000059874 1780173

[B39] Wilkins HA Blumenthal UJ Hagan P Hayes RJ Tulloch S Resistance to reinfection after treatment of urinary schistosomiasis Trans R Soc Trop Med Hyg 1987 81 29 35 3127957 10.1016/0035-9203(87)90273-2

[B40] Demeure CE Rihet P Abel L Ouattara M Bourgois A Dessein AJ Resistance to *Schistosoma mansoni *in humans- influence of the IgE/IgG4 balance and IgG2 in immunity to reinfection after chemotherapy J Infect Dis 1993 168 1000 1008 10.1093/infdis/168.4.1000 7690821

[B41] Southgate V Tchuem Tchuenté LA Sène M De Clercq D Théron A Jourdane J Webster BL Rollinson D Grysels B Vercruysse J Studies on the biology of schistosomiasis with emphasis on the Senegal River Basin Mem Inst Oswaldo Cruz 2001 96 75 78 11586429 10.1590/s0074-02762001000900010

[B42] Butterworth AE Fulford AJ Dunne DW Ouma JH Sturrock RF Longitudinal studies on human schistosomiasis Philos Trans R Soc Lond B Biol Sci 1988 321 495 511 10.1098/rstb.1988.0105 2907155

[B43] Hagan P Reinfection, exposure and immunity in human schistosomiasis Parasitol Today 1992 8 12 16 10.1016/0169-4758(92)90303-J 15463518

[B44] Kloos H Gazzinelli A Van Zuyle P Microgeographical patterns of schistosomiasis and water contact behaviour; examples from Africa and Brazil Mem Inst Oswaldo Cruz 1998 93 37 50 9921322 10.1590/s0074-02761998000700006

[B45] Fulford AJ Webster M Ouma JH Kimani G Dunne DW Puberty and age-related changes in susceptibility to schistosome Infection Parasitol Today 1998 14 23 26 10.1016/S0169-4758(97)01168-X 17040685

[B46] Butterworth AE Dalton PR Dunne DW Mugambi M Ouma JH Richardson BA Arap Siongok TK Sturrock RF Immunity after treatment of human schistosomiasis mansoni. I. Study design, pretreatment observations and the results of treatment Trans R Soc Trop Med Hyg 1984 78 108 123 10.1016/0035-9203(84)90190-1 6710563

[B47] Anderson RM May RM Helminth infections of humans: mathematical models, population dynamics, and control Adv Parasitol 1985 24 1 101 3904343 10.1016/s0065-308x(08)60561-8

[B48] Cheever AW Kamel IA Elwi AM Mosimann JE Danner R *Schistosoma mansoni *and *S. haematobium *infections in Egypt. II Quantitative parasitological findings at necropsy Am J Trop Med Hyg 1977 26 702 716 889013 10.4269/ajtmh.1977.26.702

[B49] Medley G Anderson RM Density-dependent fecundity in *Schistosoma mansoni *infections in man Trans R Soc Trop Med Hyg 1985 79 532 534 10.1016/0035-9203(85)90087-2 3936242

[B50] Cheever AW Density-dependent fecundity in *Schistosoma mansoni *infections in man: a reply Trans R Soc Trop Med Hyg 1986 80 991 992 3111033 10.1016/0035-9203(86)90284-1

[B51] Wertheimer SP Vermund SH Lumey LH Singer B Lack of demonstrable density-dependent fecundity of schistosomiasis mansoni: analyses of Egyption quantitative human autopsies Am J Trop Med Hyg 1987 37 79 84 3111282 10.4269/ajtmh.1987.37.79

[B52] Gryseels B De Vlas SJ Worm burdens in schistosome infections Parasitol Today 1996 12 115 119 10.1016/0169-4758(96)80671-5 15275241

[B53] Polman K De Vlas SJ Gryseels B Deelder AM Relating serum circulating anodic antigens to faecal egg counts in *Schistosoma mansoni *infections: a modelling approach Parasitology 2000 121 601 610 11155931 10.1017/s0031182000006843

[B54] Polman K De Vlas SJ Van Lieshout L Deelder AM Gryseels B Evaluation of density-dependent fecundity in human *Schistosoma mansoni *infections by relating egg counts to circulating antigens through Deming regression Parasitology 2001 122 161 167 11272646 10.1017/s0031182001007193

[B55] Friedman JF Kurtis JD McGarvey ST Fraga AL Silveira A Pizziolo V Gazzinelli G LoVerde P Corréa-Oliveira R Comparison of self-reported and observed water contact in an *S. mansoni *endemic village in Brazil Acta Trop 2001 78 251 259 10.1016/S0001-706X(01)00094-8 11311188

[B56] Payne G Carabin H Tallo V Alday P Gonzalez R Joseph L Olveda R McGarvey ST Concurrent comparison of three water contact measurement tools in four endemic villages of the Philippines. The schistosomiasis transmission ecology in the Philippines project (STEP) Trop Med Int Health 2006 11 834 842 10.1111/j.1365-3156.2006.01638.x 16772005

[B57] Hagan P Wilkins HA Blumenthal UJ Hayes RJ Greenwood BM Eosinophilia and resistance to *Schistosoma haematobium *in man Parasite Immunol 1985 7 625 632 10.1111/j.1365-3024.1985.tb00106.x 3937975

[B58] Sama MT Ratard RC Water contact and schistosomiasis infection in Kumba, south-western Cameroon Ann Trop Med Parasitol 1994 88 629 634 7893177 10.1080/00034983.1994.11812914

[B59] Bethony J Williams JT Brooker S Gazzinelli A Gazzinelli MF LoVerde PT Corréa-Oliveira R Kloos H Exposure to *Schistosoma mansoni *infection in a rural area in Brazil. Part III: household aggregation of water-contact behaviour Trop Med Int Health 2004 9 381 389 10.1111/j.1365-3156.2004.01203.x 14996368

[B60] Sow S Polman K Vereecken K Vercruysse J Gryseels B De Vlas SJ The role of hygienic bathing after defecation in the transmission of *Schistosoma mansoni* Trans R Soc Trop Med Hyg 2008 102 542 547 10.1016/j.trstmh.2008.02.016 18423504

